# An Analysis of Infectious Failures in Acute Cholangitis

**DOI:** 10.1155/1994/73139

**Published:** 1994

**Authors:** Jesse Thompson, Robert S. Bennion, Henry A. Pitt

**Affiliations:** 1 Departments of Surgery Olive View/UCLA Medical Center UCLA School of Medicine The Johns Hopkins Hospital USA; 2 Department of Surgery Olive View/UCLA Medical Center, 2B156 14445 Olive View Drive Sylmar California 91342 USA

## Abstract

To determine the factors responsible for therapeutic failures in acute cholangitis, a series of 127 patients was analyzed. There were 64 females and 63 males whose mean age was 57.2 years. Ninetyfour (74.0%) of these patients were clinically cured with initial measures, whereas 33 patients (26%) failed initial therapy for an infectious reason. No differences were observed between the two groups in regard to age and gender. However, more patients in the group that failed had a malignant cause for their bile duct obstruction (72.7% vs. 42.6%, p < 0.01) and had a pretreatment positive blood culture (45.5% vs. 13.8%, p < 0.01). Patients who failed had a higher mean total bilirubin level (9.7 mg/dl vs. 5.5 mg/dl, p < 0.005) and more of them had a level greater than 2.2 mg/dl (97% vs. 69.9%, p < 0.001). Also, more bile cultures were initially positive (93.9% vs. 76.6%, p < 0.05) and more organisms were isolated per culture (3.88 vs. 2.86, p < 0.03) in the patients who failed. In addition, more patients failed who had two or more organisms in the bile (33% vs. 8.3%, p < 0.02). Patients in whom *Candida*, or any panresistant organism was isolated also tended to fail. Multivariant analysis showed that malignancy, bacteremia, bilirubin ≥ 2.2 mg/dl, ≥ 2 organisms in the bile and a panresistant organism were the best predictors of treatment failure with a serum bilirubin level ≥ 2.2 mg/dl being the variable that increases a patient's log-odds ratio of failure the greatest. In conclusion, patients with acute cholangitis who have an increased chance to fail initial therapy can be identified, and treatment altered accordingly.


*HPB Surgery*, 1994, Vol. 8, pp. 139–145
Reprints available directly from the publisher
Photocopying permitted by license only

© 1994 Harwood Academic Publishers GmbH
Printed in Malaysia

### An Analysis of Infectious Failures in Acute Cholangitis

JESSE THOMPSON[1], ROBERT S. BENNION[2] and HENRY A. PITT[3]

Departments of Surgery, Olive View/UCLA Medical Center, UCLA School of Medicine, and The Johns Hopkins Hospital

To determine the factors responsible for therapeutic failures in acute cholangitis, a series of 127 patients was analyzed. There were 64 females and 63 males whose mean age was 57.2 years. Ninety-four (74.0%) of these patients were clinically cured with initial measures, whereas 33 patients (26%) failed initial therapy for an infectious reason. No differences were observed between the two groups in regard to age and gender. However, more patients in the group that failed had a malignant cause for their bile duct obstruction (72.7% vs. 42.6%, $p < 0.01$) and had a pretreatment positive blood culture (45.5% vs. 13.8%, $p < 0.01$). Patients who failed had a higher mean total bilirubin level (9.7 mg/dl vs. 5.5 mg/dl, $p < 0.005$) and more of them had a level greater than 2.2 mg/dl (97% vs. 69.9%, $p < 0.001$). Also, more bile cultures were initially positive (93.9% vs. 76.6%, $p < 0.05$) and more organisms were isolated per culture (3.88 vs. 2.86, $p < 0.03$) in the patients who failed. In addition, more patients failed who had two or more organisms in the bile (33% vs. 8.3%, $p < 0.02$). Patients in whom *Candida*, or any panresistant organism was isolated also tended to fail. Multivariant analysis showed that malignancy, bacteremia, bilirubin $\geq 2.2$ mg/dl, $\geq 2$ organisms in the bile and a panresistant organism were the best predictors of treatment failure with a serum bilirubin level $\geq 2.2$ mg/dl being the variable that increases a patient's log-odds ratio of failure the greatest. In conclusion, patients with acute cholangitis who have an increased chance to fail initial therapy can be identified, and treatment altered accordingly.

KEY WORDS: Cholangitis   biliary infection   biliary antibiotics

Acute cholangitis is infection in an obstructed biliary system from both benign and malignant causes. Initial therapy is directed toward general support of the patient including fluid and electrolyte replacement, correction of metabolic derangements, broad spectrum antibiotic therapy, and biliary drainage. The majority of patients with acute cholangitis are cured of their infection by these efforts. However, there are patients in whom this early treatment of cholangitis fails and who require alteration in therapy, such as a change in antibiotics, further biliary drainage, or treatment of an intervening infection like a wound infection or pneumonia. The factors involved in these infectious failures are not completely clear, and the purpose of this study is to analyze a series of patients with acute cholangitis to elucidate those elements that play a role in patients who fail initial therapy for an infectious cause.

*Address for correspondence:* Jesse Thompson, M.D., Department of Surgery, Olive View /UCLA Medical Center, 2B156, 14445 Olive View Drive, Sylmar, California 91342.

### PATIENTS AND METHODS

### *Patient Selection*

The patients were selected from among those with acute cholangitis treated at the University of California at Los Angeles Medical Center and the Olive View/UCLA Medical Center in Los Angeles, California, and the Johns Hopkins Medical Center in Baltimore, Maryland, in a prospective fashion on two consecutive antibiotic protocols from 1984 to 1988. The antibiotic regimens employed were either a penicillin/aminoglycoside combination or a broad spectrum penicillin, both of which have been proven effective in treating acute cholangitis[1–3]. The inclusion criteria were a clinical diagnosis of acute cholangitis supported by either pain or tenderness in the right upper quadrant or jaundice, and hypothermia of less than 36.5 degrees C.; fever of more than 38.0 degrees C.; septic shock; or white blood cell (WBC) count of more than $10 \times 10^9$/L. Patients were not included if another medical or surgical illness could explain the illness. All patients included in this study had obstruction of the biliary tract documented roentgenographically and/or at surgery.

### Patient Evaluation

Pretreatment blood cultures were obtained from all patients. Aerobic and anaerobic bile cultures were obtained from indwelling biliary tubes at the time of percutaneous draining, or at surgery and were transported to the microbiology laboratory in anaerobic transport media. All antibiotics were given intravenously for a minimum of five days unless a clinical infectious failure occurred at which time the antibiotics were usually changed.

A patient was considered a clinical cure if the signs and symptoms of cholangitis completely abated with no evidence of infection in the follow-up period. A patient was judged improved if there was a signifcant improvement in the clinical findings, but not complete resolution of the infection. The relapse category was used for patients who initially improved only to have recurrence of infection within three weeks of therapy. A failure was defined as no demonstrable response to therapy or the development of a significant postoperative infection in patients undergoing an operation. Abdominal wounds were considered infected if purulent material that subsequently grew organisms on culture could be obtained from them.

### Statistical Analysis

Student's two-tailed t-test and the chi-square test were used to compare the differences between the study groups and treatment results. Logistic regression analysis was employed to evaluate the relative importance of the differences observed between the study groups for the individual variables. The data are expressed as mean (plus or minus standard error of the mean) or as a percent of patients.

### RESULTS

### Demographics

There were 127 patients with acute cholangitis eligible for analysis. The number of patients in the improved and relapse categories was too small for valid statistical analysis, and these patients were included in the clinical cure and failure groups, respectively. Therefore, 94 patients (74%) had a clinical cure and 33 (26%) were judged to be a clinical failure. The reasons for failure were persistent sepsis in 28 patients (85%) and a wound infection in five patients (15%).

The mean age of the patients in this series was 57.2 years, and there were 64 females (50.4%) and 63 males (49.6%). There were no differences between the cure and failure groups with regard to age or gender. In the cured group the mean age was 56.8 ± 1.5 years and there were 47 females (50%) compared to a mean age of 58.4 ± 1.9 years and 17 females (51.5%) in the failure group. No difference was observed in the admitting temperature between the two groups (38.5 ± 0.08° C in those patients who were cured vs. 38.8 ± 0.15 °C in those who failed).

Sixty-three patients in this series had a benign cause for their bile duct obstruction (stones-43, stricture-15, sclerosing cholangitis -3, other -2), and 64 had a malignant etiology (bile duct cancer - 41, pancreatic cancer - 11, colon cancer-4, gallbladder cancer -3, other-5). Nearly three-fourths (24/33) of the failure patients had a malignant obstruction compared to only 43% (40/94) of the cured patients ($p < 0.01$).

The comorbidities present in the patients in this series are listed in Table 1. The hematopoietic disease observed in these patients was generally chronic anemia although one patient in the failure group had hypersplenism and one in the cured group had polycythemia vera. Patients were considered to have cardiac disease if they had underlying coronary artery disease, valvular heart disease or congestive heart failure and hepatic disease if they had documented cirrhosis or hepatitis. The renal disease refers to preexisting chronic renal failure. No differences were noted between the groups as 72 patients (76.6%) in the cured group had a major comorbidity, while 23 patients (69.7%) in the failure group were so afflicted.

Three patients in this series presented in septic shock, 2 (6%) in the group that failed and 1 (1%) in the group that was cured. In addition 3 patients (11%), all in the failure group, had mild acute reversible renal failure associated with their attack of acute cholangitis. The finding was significant ($p < 0.03$).

### Laboratory Data

The laboratory data is shown in Table 2. The two groups were similar with respect to initial white blood cell count,

**Table 1** Major comorbidities in patients with acute cholangitis

| | *Cured (N=94)* | *Failure (N=33)* |
|---|---|---|
| Hypertension | 19 (20.2%) | 10 (30.3%) |
| Hematopoietic | 14 (14.9%) | 4 (12.1%) |
| Diabetes | 14 (14.9%) | 3 (9.1%) |
| Cardiac | 13 (13.8%) | 7 (21.2%) |
| Pulmonary | 12 (12.8%) | 2 (6.1%) |
| Hepatic | 8 (8.5%) | 4 (12.1%) |
| Renal | 5 (5.3%) | 0 |
| Total | 72 (76.6%) | 23 (69.7%) |

alkaline phosphatase, SGOT, SGPT, creatinine, and BUN. The only significant difference observed was a higher total bilirubin level in the failure group (9.7 ± 1.3 mg/dl vs. 5.5 ± 0.5 mg/dl, $p < 0.005$). In addition, more patients in the group that failed had a total bilirubin level greater than 2.2 mg/dl (97% vs. 69.9%, $p < 0.001$).

### *Operations and Procedures*

In addition to antibiotic treatment, biliary drainage was liberally employed in the patients in this series. Biliary drainage tubes, primarily of the transhepatic type, were the most common modality employed, and were used to stent anastomoses and palliate malignancies as well as to drain infected bile. More patients in the failure group had tube drainage compared to those that were cured (90.9% vs. 60.6%, $p < 0.001$). The vast majority of these tubes were already present prior to the attack of acute cholangitis, and treatment included irrigation, replacement, and repositioning. There were only two patients in the failure group in whom complete drainage of the biliary tract was probably not accomplished, and both had an unresectable malignancy.

Concerning operations and other types of invasive procedures exclusive of biliary tube drainage, no differences were noted between the two study groups. In the group that was cured, 50% of patients had such a procedure compared to 51.5% of those who failed. The specific procedures are shown in Table 3.

### *Bacteriology*

Twenty-eight patients (22%) in this series had a positive blood culture associated with their attack of acute cholangitis, and this bacteremia occurred more frequently in the failure patients (45.5% vs. 13.8%, $p < 0.01$). In most instances, the same organism found in the blood was eventually recovered from the bile. In addition, 103 patients (81.1%) had bactibilia on the initial bile culture, and the failure patients were more likely than those who were cured to have a positive culture (93.9% vs. 76.6%, $p < 0.05$). The patients who failed also had more organisms per specimen (3.88 vs. 2.86, $p < 0.03$), and more of them had two or more organisms in the bile (33% vs. 8.3%, $p < 0.02$).

The actual organisms cultured from the bile in the two groups of patients are shown in Table 4. There was a tendency for *Enterobacter*, *Streptococcus sp*, and *Candida* to be present more often in the failure patients, but this tendency was significant only for *Candida* (27.3% vs. 10.6%, $p < 0.05$). Other types of yeast were isolated occasionally in this series, and all yeast as a group was also present significantly more often in the patients who failed (33% vs. 11.7%, $p < 0.02$).

No pure cultures of anaerobic organisms were isolated in this series of patients, and there was the same percentage of mixed aerobic/anaerobic cultures in the two groups (18.2% in the failure patients and 17.0% in the cured patients). Most patients had pure aerobic cultures. Eight patients, each with at least three bacteria in their bile culture, were found to have an organism resistant to all antibiotics tested for. Five of these patients failed, three were cured (15.2% vs. 3.2%, $p < 0.05$), and all had a malignant etiology. Lastly, no particular combinations of

**Table 2** Laboratory data in patients with acute cholangitis

| | *Cured* (*N=94*) | *Failure* (*N=33*) |
|---|---|---|
| WBC ($10^9$/L | 14.0 ± 0.7 | 14.6 ± 1.5 |
| Bilirubin (MG/DL) | 5.5 ± 0.5* | 9.7 ± 1.3* |
| Alk Phos (IU) | 481.6 ± 38.8 | 491.4 ± 47.2 |
| SGOT (IU) | 130.4 ± 20.2 | 88.4 ± 9.9 |
| SGPT (IU) | 136.6 ± 21.4 | 92.3 ± 10.4 |
| BUN (MG/DL) | 13.6 ± 0.8 | 15.0 ± 1.8 |
| Creatinine (MG/DL) | 1.0 ± 0.03 | 1.1 ± 0.1 |
| | | p < 0.005 |

**Table 3** Operations and procedures in patients with acute cholangitis

| | *Cured* (*N=94*) | *Failure* (*N=33*) |
|---|---|---|
| Cholecystectomy, CBDE | 20 (21.3%) | 2 (6.1%) |
| Cholecystectomy | 9 (9.6%) | 2 (6.1%) |
| Choledochojejunostomy | 7 (7.4%) | 5 (15.2%) |
| ERCP-/-Sphincterotomy | 4 (4.3%) | 1 (3.0%) |
| Percutaneous Drain Abscess | 4 (4.3%) | 1 (3.0%) |
| Choledochoduodenostomy | 2 (2.1%) | 1 (3.0%) |
| Exploratory Laparotomy | 1 (1.1%) | 4 (12.1%) |
| Whipple Procedure | 0 | 1 (3.0%) |

**Table 4** Organisms recovered from the bile in patients with acute cholangitis

| | *Cured* (*N=94*) | *Failure* (*N=33*) |
|---|---|---|
| E. coli | 39.3% | 48.5% |
| Enterococcus | 45.7% | 42.4% |
| Klebsiella | 39.3% | 36.4% |
| Enterobacter | 21.3% | 33.3% |
| Streptococcus | 11.7% | 30.3% |
| Pseudomonas | 21.3% | 27.3% |
| Candida | 10.6%* | 27.3%* |
| Citrobacter | 14.9% | 18.2% |
| Bacteroides | 11.7% | 12.1% |
| Clostridia | 5.3% | 12.1% |
| | | * p < 0.005 |

organisms were isolated more frequently in the failure patients.

Most of the patients who failed had repeat bile cultures; and as expected, there was persistence of the prevalent organisms and a tendency toward increasing antibiotic resistance, which was particularly evident with *Enterobacter* and *Pseudomonas.* Also there was a tendency to isolate yeast more often in repeat bile cultures, although this was not statistically significant (44% vs. 33%).

When patients were grouped by benign or malignant etiology of their cholangitis, it was evident that there were major differences in the organisms encountered. Specifically, *Pseudomonas sp, Enterobacter sp, Enterococcus sp, Klebsiella sp,* and all yeast were encountered significantly more frequently in patients with malignancies ($p < 0.005$ for each). It should be noted that only one patient with a benign cause for cholangitis had yeast on the initial culture ($p < 0.00001$).

### *Deaths and Complications*

Five patients (4%) in this series died during the hospitalization for acute cholangitis, and each had a malignant bile duct obstruction. No significant difference in mortality was noted between the patients who were cured and those who failed (3.2% vs. 6.1%). Hepatic failure was a factor in the death of all five patients.

The major complications encountered in this series were transient renal toxicity and hematopoietic toxicity, which was manifest as an elevated prothrombin or partial thromboplastin time. A greater percentage of patients in the failure group developed a complication (21.2% vs. 9.6%) as well as renal (12.1% vs. 6.4%) or hematopoietic (15.2% vs. 5.3%), but these differences were not significant.

Five patients in this series had liver abscesses, and there were no differences between the groups, as four of these patients (4.3%) were cured and one (3%) was a failure.

### *Multivariant Analysis*

The results reported above suggest that the single variables of malignancy, positive blood culture, acute renal failure, bactibilia, multiple organisms in the bile, yeast and panresistant organisms in the bile, elevated bilirubin level, and biliary drainage tubes are risk factors influencing treatment failure in patients with acute cholangitis. Multivariant analysis was performed using logistic regression to analyze the relative significance of these variables as risk factors both independently and in combination. It was found that various combinations of positive blood culture, malignancy, bilirubin ≥ 2.2 mg/dl, multiple ( ≥ 2) organisms in the bile, and panresistant organisms in the bile are the best predictors of treatment failure. In addition, a serum bilirubin level ≥ 2.2 mg/dl is the one variable that increases an individual patient's log-odds ratio of failure the greatest, when all the other variables are corrected for. These log-odds ratios for all five parameters are shown in Table 5.

**Table 5** Odds ratio of failure in patients with acute cholangitis

| *Parameter* | *Odds Ratio* |
|---|---|
| Malignanacy | 1.93 |
| Panresistant organism | 3.55 |
| Bacteria ≥ 2 | 5.93 |
| Bacteremia | 8.14 |
| Elevated Bilirubin | 30.11 |

Applying the mathematical model for the parameters of malignancy, positive blood culture, bilirubin level ≥ 2.2 mg/dl, and multiple ( ≥ 2) organisms in the bile produces the predicted probabilities for failure exhibited in Table 6. The presence of panresistant organisms is purposely excluded as this information is generally not available within a time frame to be clinically useful. As shown, if all four parameters are absent, there is a 4% predicated probability of failure, whereas when all four are present, there is a greater than 99% predicted probability of failure. In addition, bilirubin level alone is more predictive of failure than malignancy alone or combined with either positive blood culture or multiple organisms in the bile.

### DISCUSSION

The purpose of this review was to identify in a retrospective fashion those factors involved in infectious treatment failures in patients with acute cholangitis. Of note, three of the five most significant variables identified by multivariant analysis, i.e. positive blood

**Table 6** Probability of failure in patients with acute cholangitis

| *Malignancy* | *Bacteremia* | *Elevated Bilirubin* | *Bacteria ≥2* | *% Failure* |
|---|---|---|---|---|
| — | — | — | — | 4.0 |
| + | — | — | — | 8.3 |
| — | — | — | + | 21.4 |
| — | + | — | — | 24.6 |
| + | — | — | + | 37.1 |
| + | + | — | — | 41.4 |
| — | — | + | — | 52.8 |
| — | + | — | + | 68.0 |
| + | — | + | — | 70.7 |
| + | + | — | + | 82.1 |
| — | — | + | + | 87.9 |
| — | + | + | — | 89.7 |
| + | — | + | + | 94.0 |
| + | + | + | — | 94.9 |
| — | + | + | + | 98.3 |
| + | + | + | + | 99.2 |

culture, ≥ 2 organisms in the bile, and the presence of a panresistant organism in the bile, are infectious parameters. The white blood cell count, temperature, age and gender, which have been useful predictors of more serious illness or adverse events in some analyses,[4–6] were not discriminatory in this study.

A serum bilirubin level greater than 2.2 mg/dl was the factor that increased an individual patient's odds of failure the most. The role of jaundice or hyperbilirubinemia in increasing postoperative morbidity and mortality in patients undergoing biliary tract surgery has not been completely elucidated. Several reports suggest that an elevated bilirubin is related to the development of postoperative complications and operative mortality,[5–8] and some studies have shown that reducing the bilirubin level preoperatively in patients with obstructive jaundice can reduce the incidence of postoperative complications and death[9,10]. Other reports, though, have shown that preoperative reduction of the bilirubin level has no impact on postoperative problems[11,12]. In addition, it is suggested in one study that jaundice per se does not contribute substantially to an undesirable postoperative outcome[13]. In this study, the determinant of poor outcome was an infectious failure following initial therapeutic measures for acute cholangitis. It is known that patients with chronic obstructive jaundice have impaired neutrophil functions and other host defense mechanisms such as synthesis of acute-phase proteins and complement[14], and conceivably these deficits were operative in some patients in this series.

A malignant etiology for bile duct obstruction is another major variable that increased the chance of failure in this study. There seems to be rather universal agreement that malignancy correlates with poor results in patients with obstructive jaundice who have surgery, with or without infection[5–7,13,15]. It is also well recognized that both cellular and humoral immunity are impaired either because of the underlying malignancy and/or the antineoplastic therapy[16]. In addition, malnutrition is a frequent complication of malignancy which further decreases host defense. This level of immunocompromise in patients with cancer makes them more susceptible to infectious complications such as acute cholangitis, and makes the treatment of these problems all the more difficult.

Bacteremia is a frequent occurrence in patients with acute cholangitis as evidenced by the 22% incidence in our patients. It has been shown that obstructive jaundice can promote bacterial translocation from the gut in an animal model, possibly by an impairment in the mucosal barrier function, as well as by impaired host immune defenses[17]. It is not known if this leads to bacteremia or bactibilia or both. The bacteremia may also arise from an obstructed, colonized bile duct. Since dissemination of microorganisms into the circulation can result when local host defense mechanisms fail anywhere[18], bacteremia is a natural consequence of acute cholangitis in many patients. It is not surprising that bacteremia was a significant predictor of infectious failure in our patients. Dissemination of microbes via the blood stream will activate the systemic inflammatory response which can be a potential host liability if multiorgan failure intervenes[18].

Most patients in this series had bactibilia. The organisms isolated are similar to those that have been reported by others[19–22]. Like these reports, multiple organisms were isolated from the bile in the majority of the patients in this study. However, those patients in whom the bile cultures grew two or more organisms were at greater risk of failure than those patients whose cultures grew none or, at most, one organism. Since a sterile bile culture could be interpreted as eradication of infection, one would expect these patients to have less infectious sequelae following adequate therapy. Moreover, the presence of two or more organisms might be an indication of a greater infectious burden or bacterial synergy, both of which are more difficult to treat, especially in patients who are jaundiced and/or have an underlying malignancy.

The only organisms that were present significantly more often in the patients who failed were panresistant ones and yeast. In our experience complete eradication of all the organisms in the bile is uncommon, even in those patients who are cured of their acute infectious illness. Complete eradication would be particularly difficult to achieve for a patient with either a panresistant organism or a tenacious opportunistic pathogen such as yeast. A bile culture containing a panresistant organism was one of the major determinants of failure in the multivariant analysis. It should be noted that *Enterococcus* was present in the bile culture of the failure patients at least as often as the other major pathogens such as *E. coli* and *Klebsiella*, but its significance regarding predilection for failure was not apparent. Whether *Enterococcus* is a major pathogen in non-nosocomial intra-abdominal infection has not been completely elucidated[23], and the results of the current study do not help to clarify this question.

Biliary drainage tubes were present in many of the patients in this series and were used for a variety of indications, including drainage of infected bile and stenting of unresectable malignancies or tenuous anastomoses. Many times in this series occlusion or some other malfunction of the tube was the initiating event in the development of an attack of acute cholangitis. Irrigation, repositioning, or even replacement was often required to control the infection. Even though the presence of a biliary tube was not one of the most important factors influencing an infectious failure following initial treatment of acute cholangitis, tubes in general do complicate matters. First, they are a foreign body generally draining to the outside providing easy access of flora into the biliary tract and making complete eradication impossible, and second, they precipitate repeat attacks of acute cholangitis in some patients who then receive numerous courses of antibiotics[15]. In addition, some clinicians place these patients on long-term suppressive oral antibiotic therapy which can lead to the development of resistant organisms and the emergence of opportunistic pathogens, particularly yeast.

Complications of therapy and death were somewhat more prevalent in those patients who were infectious failure in this series than in those who were cured, but these differences were not significant. One would expect patients with more serious infections to have more problems, but this trend could not be fully documented in this report.

### SUMMARY

A series of patients with acute cholangitis has been analyzed. For those patients who do not respond to initial therapy, several risk factors have been identified. The most important of these are a serum bilirubin greater than 2.2 mg/dl, bacteremia, a malignant cause of the bile duct obstruction, two or more organisms in the bile culture, and a panresistant organism in the bile. Most of these parameters are generally known within the first 48 to 72 hours of therapy. Therefore, early in the course of treatment, the clinician should be alerted to the possibility that the patient with two or more of these risk factors has a greater chance to fail, and be prepared to alter the treatment plan. Such alterations should include broadening or changing the antibiotic coverage, increased diligence in monitoring for signs of impending sepsis, and assuring adequate and complete drainage of the biliary tract.

### REFERENCES

1. Muller, E. L., Pitt, H. A., Thompson, J. E., Jr., *et al.* (1987) Antibiotics in infections of the biliary tract. *Surg Gynecol Obstet* **165,** 285–292.
2. Thompson, J. E., Jr., Pitt, H. A., Doty, J. E., *et al.* (1990) Broad spectrum penicillin as an adequate therapy for acute cholangitis. *Surg Gynecol Obstet* **171,** 275–282.
3. Gerecht, W. B., Henry, H. K., Hoffman, W. W., *et al.* (1989) Prospective randomized comparison of mezlocillin therapy alone with combined ampicillin and gentamicin therapy for patients with cholangitis. *Arch Int Med* **149,** 1279–1284.
4. Thompson, J. E., Jr., Bennion, R. S., Doty, J. E., *et al.* (1990) Predictive factors for bactibilia in acute cholecystitis. *Arch Surg* **125,** 261–264.
5. Pitt, H. A., Cameron, J. L., Postier, R. G., and Gadacz, T. R., (1981) Factors affecting mortality in biliary tract surgery. *Am J Surg* **141,** 66–72.
6. Gigot, J. F., Leese, T., Dereme, T. *et al.* (1989) Acute cholangitis: Multivariate analysis of risk factors. *Ann Surg* **209,** 435–438.
7. Dixon, J. M., Armstrong, C. P., Duffy, S. W., and Davies, G. C., (1983) Factors affecting morbidity and mortality after surgery for obstructive jaundice. *Gut* **24,** 845–852.
8. Blamey, S. L., Fearon, K. C. H., Gilmour, W. H. *et al.* (1983) Prediction of risk in biliary surgery. *Br J Surg* **70,** 535–538.
9. Denning, D. A., Ellison, E. C., and Carey, L. C., (1981) Preoperative percutaneous transhepatic biliary decompression lowers operative morbidity in patients with obstructive jaundice. *Am J Surg* **141,** 61–65.
10. Gundry, S. T., Strodel, W. E., Knol, J. A., *et al.* (1984) Efficacy of preoperative biliary tract decompression in patients with obstructive jaundice. *Arch Surg* **119,** 703–08.
11. Hatfield, A. R. W., Tobias, R., Terblanche, J., *et al.* (1982) Preoperative external biliary drainage in obstructive jaundice. *Lancet* **ii,** 896–899.
12. Pitt, H. A., Gomes, A. S., Lois, J. F., *et al.* (1985) Does preoperative biliary drainage reduce operative risk or increase hospital cost? *Ann Surg* **201,** 545–553.
13. Pellegrini, C. A., Allegra, P., Bongard, F. S., and Way, L. W. (1987) Risk of biliary surgery in patients with hyperbilirubinemia. *Am J Surg* **154,** 111–117.
14. Fisher, J. R., Cirrhosis and Jaudice. In: Wilmore, D. W., Brennan, M. F., Harken, A. H., Holcroft, J. W., Meakins, J. L., eds. (1989) The American College of Surgeons Care of the Surgical Patient. New York: Scientific American, Section VII, Chapter 5.
15. Thompson, J. E., Jr., Tompkins, R. K., and Longmire, W. P., Jr. (1982) Factors in management of acute cholangitis. *Ann Surg* **195,** 137–145.
16. Pizzo, P. A., and Meyers, J. (1989) Infections in the cancer patient. In: Devita, V T., Helman, S., Rosenberg, S A., eds. (1989) Cancer: Principles and Practice of Oncology. Philadelphia: J. B., Lippicott, 2088–2133.
17. Deitch, E A., Sittig, K., Li., M., Berg, R., and Specian, R. D. (1990) Obstructive jaundice promotes bacterial translocation from the gut. *Am J Surg* **159,** 79–84.
18. Fry, D. E. The positive blood culture. In: Wilmore, D. W., Brennan, M. F., Harken, A. H., Holcroft, J.W., Meakins, J. L., eds. (1988) The American College of Surgeons Care of the Surgical Patient. New York: Scientific American, Section IX, Chapter 2.
19. Szabo, S., Mendelson, M. H., Mitty, H. A., *et al.* (1987) Infections associated with transhepatic biliary drainage devices. *Am J Med* **82,** 921–926.
20. Khardori, N., Wong, E., Carrasco, C. H., *et al.* (1991) Infections associated with biliary drainage procedures. *Rec Infect Dis* **13,** 587–591.
21. Levine, J. G., Botet, J., and Kurtz, R., (1990) Microbiological analysis of sepsis complicating non-surgical biliary drainage in malignant obstruction. *Gastrointest Endosc* **36,** 364–368.
22. Lipsett, P. A., and Pitt, H. A., (1990) Acute cholangitis. *Surg Clin North Am* **70,** 1297–1312.
23. Bennion, R. S., (1991) Enterococcus in the surgical patient. *Postgrd Gen Surg* **3,** 168–169.

### INVITED COMMENTARY

### An Analysis Of Infectious Failures In Acute Cholangitis

Acute cholangitis is still associated with a substantial morbidity and mortality despite improvements in modern antibiotic therapy and intensive care treatment. The identification of factors predisposing to treatment failure should thus of great clinical value. The present study demonstrates that high bilirubin levels, positive bile cultures, multiple bacterial growth, positive blood cultures prior to treatment and malignancy correlate with a higher risk of treatment failure in acute cholangitis. The finding of increased bilirubin level, reported to the most important factor, could have several explanations. Biliary obstruction induces a variety of alterations in host defense, such as an impairment in RES function particularly Kupffer cell function[1-3]. This could be due to factors like increased intraductal pressure, increased plasma concentrations of antimetabolic substances or depletion of serum opsonins otherwise promoting phagocytosis. Furthermore, increased bilirubin levels by inhibiting cellular respiratory enzymes might impair energy dependent phagocytosis[4]. The endotoxemia noted in patients with obstructive jaundice probably also has an influence, e.g. by inducing macrophage dysfunction[5,6] and the lack of intestinal luminal bile salts in biliary obstruction also increases endotoxemia as bile salts possess a direct detergent effect on endotoxin[7]. The presence of biliary obstruction might also constitute a hindrance to hepatocyte excretion and it might thus be more difficult to obtain the required concentrations of antibiotics in bile.

The present study demonstrates bactibilia as a factor predisposing to treatment failure. In patients with common bile duct stones and acute suppurative cholangitis, bacterial cultures from bile seem to be always positive[8,9]. It is to be emphasized that anaerobic bacteria, mainly *Clostridium* and *Bacteroides,* with improved culture techniques are demonstrated in maybe 20–30% of positive cultures[10,11]. Thus, antibiotics also covering the anaerobic flora should be considered. Otherwise, as also demonstrated in the present study, bacterial culture findings fairly well represent bacteria present in the distant small intestine[12,13]. The use of biliary drainage tubes tended, though not significantly, to predispose to treatment failures. Septic events following both percutaneous transhepatic drainage and endoscopic biliary drainage are frequent[14,15] and reported factors predisposing to the development of cholangitis following the use of biliary drainage are among others malignancy, duration of biliary drainage and incomplete bile drainage[14,16]. In order to improve the outcome of patients with acute cholangitis, the definition of factors predisposing to treatment failure are of great clinical usefulness. Patients at high risk need special consideration and might qualify for more intense and extensive treatment from the very beginning.

### REFERENCES

1. Drivas, G., James, O., and Wardle, N. (1976) Study of reticuloendothelial phagocytic capacity in patients with cholestasis. *Br Med J* **1**, 1568–1569.
2. Scott-Connor, C. E. M., Grogan, J. B., Scher, K. S., and Bernstein, J. (1989) Impaired clearance of *Escherichia coli* bacteremia in early biliary obstruction. *Am J Surg* **157**, 210–214.
3. Ding, J. W., Andersson, R., Norgren, L., Stenram, U., Bengmark, S. (1992) Influence of biliary obstruction and sepsis on reticuloendothelial function in rats. *Eur J Surg* **158**, 157–164.
4. Cowger, M. L., Igo, R. P., Labbe, R. F. (1965) The mechanism of bilirubin toxicity studied with purified respiratory enzyme and tissue cultural system. Biochemistry **4**, 2763–2770.
5. Heilman, D. H. (1977) Regulation of endotoxin-induced inhibition of macrophage migration by fresh serum. *Inf Immun* **17**, 371–377.
6. Christman, J. V., Petras, S. F., Hacker, M., Abscher, P. M., Davis, G. S. (1988) Alveolar of macrophage function is selectively altered after endotoxemia in rats. *Inf Immun* **56**, 1254–1259.
7. Shands, J. W. Jr., Chun, P. W. (1980) Dispersion of gram negative lipopolysaccharide by deoxycolate. *J Biol Chem* **255**, 1221–1226.
8. Maluenda, F., Csendes, A., Burdiles, P., Diaz, J. (1989) Bacteriological study of choledochal bile in patients with common bile duct stones, with or without acute suppurative cholangitis. *Hepatogastroenterology* **36**, 132–135.
9. Lai, E. C. S., Mok, F. P. T., Tan, E. S. Y., Lo, C-M., Fan, S-T., Jou, K-T., Wong, J. (1992) Endoscopic biliary drainage for severe acute cholangitis. *New Engl J Med* **326**, 1582–1586.
10. Marne, C., Pallares, R., Martin, R., Sitges-Serra, A. (1986) Gangrenous cholecystitis and acute cholangitis associated with anaerobic bacteria in bile. *Eur J Clin* Microbiol **5**, 35–39.
11. Brook, I., (1989) Aerobic, and anaerobic microbiology of the biliary tract disease. *J Clin Microbiol* **27**, 2373–2375.
12. Siegman-Igra, U., Schwartz, D., Conforti, N., Perlok, C., Rozin, R R. (1988) Septicemia from biliary tract infection. *Arch Surg* **123**, 366–368.
13. Thompson, J. E., Bennion, R. S., Doty, J. E., Muller, E. L., Pitt, H. A. (1990) Predictive factors for bactibilia in acute cholecystitis: *Arch Surg* **125**, 261–264.
14. Deviere, J., Baiz, E. M., Detoeuf, J., Cremer, M. (1988) Long-term follow-up of patients with hilar malignant stricture treated by endoscopic internal biliary drainage. *Gastrointest Endoscop* **34**, 95–101.
15. Audisio, R. A., Bozzetti, F., Severini, A., Pellegotti, L., Bellomi, M., Cozzi, G., Pisani, P., Callegari, L., Dochi, R., Gennari, L. (1988) The occurrence of cholangitis after percutaneous biliary drainage: evaluation of some risk factors. *Surgery* **103**, 507–512.
16. Dutta, S. K., Cox, M., Williams, R. B., Eisenstat, T. E., Standiford, H. C. (1983) Prospective evaluation of the risk of bacteremia and the role of antibiotics in ERCP. *J Clin Gastroenterol* **5**, 325–329.

